# Association Between D-dimer and Early Adverse Events in Patients With Acute Type A Aortic Dissection Undergoing Arch Replacement and the Frozen Elephant Trunk Implantation: A Retrospective Cohort Study

**DOI:** 10.3389/fphys.2019.01627

**Published:** 2020-01-21

**Authors:** Tong Liu, Jun Zheng, You-Cong Zhang, Kai Zhu, Hui-Qiang Gao, Kai Zhang, Xiu-Feng Jin, Shang-Dong Xu

**Affiliations:** ^1^Department of Cardiology, Beijing Anzhen Hospital, Capital Medical University, Beijing, China; ^2^Department of Cardiac Surgery, Beijing Aortic Disease Center, Beijing Anzhen Hospital, Capital Medical University, Beijing, China

**Keywords:** D-dimer, predictor, 90-day postoperative adverse events, aortic arch replacement, frozen elephant trunk

## Abstract

**Objective:**

In the present study, we investigated the associations between D-dimer levels at admission and early adverse events in patients with acute type A aortic dissection undergoing arch replacement and the frozen elephant trunk (FET).

**Methods:**

We retrospectively analyzed data of patients with acute type A aortic dissection undergoing aortic arch surgery and FET from July 2017 to December 2018 at Beijing Anzhen Hospital. D-dimer levels were evaluated within 24 h of admission. Multivariate Cox regression analysis was used to determine independent predictors of early postoperative adverse events.

**Results:**

A total of 347 patients were included in the study. The average age of the patients was 48.07 ± 10.56 years, with male predominance (79.25%). The incidence of 90-day postoperative adverse events was 18.7%, consisting of 14.7% mortality and 4.0% permanent neurological dysfunction (PND). The median D-dimer level was 1.95 ug/ml (interquartile range, 0.77–3.16 ug/ml). Multivariable Cox regression analysis revealed that D-dimer level was independently associated with 90-day postoperative adverse events after adjustment for confounding factors (hazard ratio = 1.19 per 10 ug/ml increase in D-dimer, 95% confidence interval: 1.01–1.41; *P* = 0.039). Kaplan–Meier analysis revealed that the highest tertile (median 6.27 ug/ml) had more 90-day postoperative adverse events compared with the median and lowest tertiles (*P* = 0.0014). Sub-analysis found that the association remained unchanged.

**Conclusion:**

Increased D-dimer levels at admission were associated with 90-day postoperative adverse events in patients with acute type A aortic dissection undergoing arch replacement and FET. These results may help clinicians optimize the risk evaluation and perioperative clinical management to reduce early adverse events.

**Key Question:**

Explore the relationship between D-dimer and early outcomes in patients with aortic dissection with arch replacement.

**Key Findings:**

Increased D-dimer at admission was associated with adverse events in patients with aortic dissection with arch surgery.

**Take-Home Message:**

The high-risk patients deserve close medical monitoring.

## Introduction

The incidence of aortic dissection markedly increases with atherosclerosis and hypertension. Although surgical treatment significantly reduces the mortality of patients with aortic dissection compared with medical management, short-term mortality (30-day or in-hospital mortality) remains high (13–17%) (Mussa et al., 2016).

D-dimer, a specific degradation product of cross-linked fibrin, represents the coagulation and fibrinolytic system activation (Suzuki et al., 2009). It is now commonly used in the diagnosis of pulmonary embolism (van der Hulle et al., 2013), deep vein thrombosis (Faller et al., 2017), acute coronary syndrome (Bayes-Genis et al., 2000), and acute aortic dissection (Akutsu et al., 2005; Suzuki et al., 2009; Shao et al., 2014). For acute aortic dissection, the mechanisms of coagulation and fibrinolytic system activation might be (1) coagulant material from the aortic wall released into the circulation and (2) an accumulation of clotting factors at the site of the lesion, secondary to the local exposition of tissue factors from the torn arterial wall (Ten Cate et al., 1975). Thus, the D-dimer level is elevated in patients with acute aortic dissection. Weber et al. (2003) first observed that D-dimer levels tended to be higher in patients with acute aortic dissection, but no relation was observed between D-dimer levels and in-hospital outcome. On the other hand, Wen et al. (2013) and Huang et al. (2015) found that an elevated D-dimer level was associated with increased in-hospital mortality (Wen et al., 2013; Huang et al., 2015). Nevertheless, the sample size in these two previous studies was relatively small, and the largest sample size was 212 patients. Further, these study populations with aortic dissection not only underwent surgical treatment, but also medical treatment. In addition, few studies have evaluated the association between D-dimer levels and 90-day postoperative adverse events in patients with acute type A aortic dissection undergoing arch replacement and FET.

For these reasons, we conducted a retrospective cohort study to investigate the association between D-dimer levels and 90-day postoperative adverse events in patients undergoing arch replacement and FET using a multivariate Cox regression model containing all known associated major perioperative predictors. Our hypothesis was that the risk of 90-day postoperative adverse events would increase as D-dimer levels increased.

## Materials and Methods

### Study Design and Study Population

From July 1, 2017, to December 31, 2018, consecutive patients with aortic disease undergoing aortic arch surgery were retrospectively identified at the aortic center in the Beijing Anzhen Hospital (Capital Medical University of Beijing, China). Patients with acute type A aortic dissection who underwent total arch replacement and the frozen elephant trunk (FET) technique were recruited into this observational retrospective cohort study. Patients who (1) underwent hemi-arch replacement (*n* = 31), (2) suffered from chronic aortic disease (*n* = 69), (3) had redo sternotomy (*n* = 19), (4) did not undergo the FET technique (*n* = 40), or (5) did not have D-dimer values (*n* = 4) were excluded.

### Study Endpoint

The endpoint of this observational retrospective study was defined as early all-cause mortality or permanent neurological dysfunction (PND) during the hospital stay or within 90 days after surgery. PND was defined as the presence of permanent neurologic deficits, including abnormal movement of limbs, coma, and sensory loss affecting one side of the body, within postoperative 90-day. Confirmation of the diagnosis was made by a neurologist or by means of computed tomographic scanning or magnetic resonance imaging of the brain.

### Definition and Procedure

The type of aortic dissection was classified according to Stanford classification (Daily et al., 1970). Acute dissection was defined as clinical symptoms lasting less than 14 days (Hagan et al., 2000). Hypertension was defined by a clinical record of systolic or diastolic blood pressure greater than 140 or 90 mmHg on admission, or the use of anti-hypertension drugs. Diabetes mellitus was defined as treatment with oral hypoglycemic agents or insulin, or as having a fasting blood glucose level ≥7.0 mmol/L (126 mg/dl). Smoking status was defined as smoking within the preceding 1 year based on information in medical records. Renal insufficiency was defined as estimated glomerular filtration rate (eGFR) <60 mL/min per 1.73 m^2^, calculated using the Chronic Kidney Disease Epidemiology Collaboration (CKD-EPI) equation (Levey et al., 2009). A diagnosis of cardiac artery disease was considered if patients had a history of myocardial infarction or previous PCI or coronary artery bypass grafting (CABG) before admission. Cerebrovascular disease was defined based on relevant neurologic dysfunction before admission or previous stroke. Pericardial tamponade was diagnosed by echocardiography. The details of total arch replacement and FET implantation have been described elsewhere by our team (Sun et al., 2011; Ma et al., 2013). In brief, the arch was opened under moderate hypothermia arrest (21∼28°C). An open stent-graft was deployed into the descending aorta. The arch was replaced with a four-branched vascular graft.

### D-dimer Measurement

Within 24 h after admission, whole-blood samples were drawn into blood collection tubes containing sodium citrate (3.2%, 109 mmol/L) as the anticoagulant (9:1 ratio of blood:anticoagulant) to measure prothrombin time (PT), activated partial thromboplastin time (APTT), fibrin degradation products (FDP), and D-dimer. Venous blood was immediately sent to the clinical laboratory center of the Anzhen hospital. Plasma PT, APTT, FDP, and D-dimer were measured using the commercially available automated latex immunoturbidimetric assay (Werfen ACL TOP 700, United States) (Di Nisio et al., 2007; Wang et al., 2018). Routine blood tests and some biochemical indicators were determined by standard quantitative assay techniques, according to the manufacturers’ instructions. All assays were run in duplicate.

### Data Collected for Analysis

Clinical, operative, perfusion, and postoperative data have been retrospectively collected in a department database, and further data were extracted from operation reports, perfusion reports, intraoperative computerized records, and review of medical records. Data were compiled via the Empower Dataweb data collection management system (X&Y Solutions, Inc., Boston, MA, United States). The current study was approved by the Human Subjects Review Committee at Anzhen Hospital (Approval No. 2017058X). Follow-up data were obtained from medical records and telephone calls.

### Statistical Analysis

Categorical variables were presented as frequencies or percentages, whereas continuous variables were expressed as means ± standard deviations (normal distribution) or medians and interquartile ranges (skewed distribution). First, we grouped D-dimer levels in tertiles. The significant differences between the means and proportions of the tertiles in baseline characteristics were analyzed using a Student’s *t*-test or Mann–Whitney *U* test for continuous variables, as appropriate, and a chi-square test for categorical variables (Table 1). Second, univariate Cox regression analyses were used to evaluate the association between each significant variable and 90-day postoperative adverse events (Supplementary Table). Third, multivariate Cox regression models (Table 2) were used to examine whether D-dimer levels had an independent effect on 90-day postoperative adverse events. We simultaneously showed the results from unadjusted, minorly adjusted, and fully adjusted analyses. The covariates, when added to this model, changed the matched odds ratio by at least 10% (Kernan et al., 2000) and covariates of known clinical importance were adjusted. We expanded D-dimer levels 10 times and labeled them per 10 ug/mL change. Trend tests were based on D-dimer level tertiles as continuous variables. Fourth, survival estimates and cumulative event rates were compared using the Kaplan–Meier method by using the time-to-first event for each endpoint among D-dimer level tertiles. The log-rank test was used to compare the Kaplan–Meier hazard ratios (HR) for 90-day postoperative adverse events, and their corresponding 95% confidence intervals (CIs). Finally, subgroup analysis was done to find whether potential risk factors were influencing the results. The interactions of subgroups were inspected by multivariate adjusted Cox regression models (Table 3). All analyses were performed using Empower (R) (^1^ X&Y Solutions, Inc.) and R^2^.

**TABLE 1:** Baseline characteristics of patients according to D-dimer tertiles.

Characteristics	Total *n* = 347	Lowest tertile (T1) *n* = 116	Median tertile (T2) *n* = 115	Highest tertile (T3) *n* = 116	*P*-value
D-Dimer (ug/ml)	1.95 (0.77–3.16)	0.51 (0.33–0.77)	1.95 (1.26–2.28)	6.27 (3.16–13.27)	< 0.001
Age, *y*	48.07 ± 10.56	46.43 ± 10.69	47.43 ± 11.39	50.34 ± 9.17	0.013
Sex (men),%	275 (79.25%)	90 (77.59%)	94 (81.74%)	91 (78.45%)	0.714
BMI (kg/m2)	26.75 ± 4.35	27.00 ± 5.04	26.85 ± 4.22	26.39 ± 3.69	0.552
Smoking history	175 (51.47%)	54 (47.79%)	66 (57.39%)	55 (49.11%)	0.290
Comorbidities					
Diabetes mellitus	13 (3.75%)	3 (2.59%)	5 (4.35%)	5 (4.31%)	0.722
Hypertension	280 (80.69%)	89 (76.72%)	94 (81.74%)	97 (83.62%)	0.388
Coronary artery disease	23 (6.63%)	6 (5.17%)	8 (6.96%)	9 (7.76%)	0.720
Acute cardiac tamponade	14 (4.03%)	3 (2.59%)	2 (1.74%)	9 (7.76%)	0.073
Cerebrovascular disease	20 (5.76%)	7 (6.03%)	7 (6.09%)	6 (5.17%)	0.959
Acute visceral ischemia	8 (2.31%)	0 (0.00%)	3 (2.61%)	5 (4.31%)	0.088
Lower-extremity ischemia	26 (7.49%)	2 (1.72%)	9 (7.83%)	15 (12.93%)	0.003
Spinal cord injury	1 (0.32%)	0 (0.00%)	1 (0.94%)	0 (0.00%)	1.000
Marfan syndrome	3 (0.86%)	2 (1.72%)	1 (0.87%)	0 (0.00%)	0.552
Chronic kidney disease	58 (16.71%)	10 (8.62%)	22 (19.13%)	26 (22.41%)	0.013
LVEF%	62.49 ± 5.43	62.06 ± 6.27	62.47 ± 5.01	62.93 ± 4.91	0.492
Severe aortic regurgitation	51 (15.32%)	22 (19.82%)	14 (12.96%)	15 (13.16%)	0.502
WBC (g/L)	11.60 ± 4.06	9.53 ± 3.63	12.07 ± 4.09	13.21 ± 3.56	< 0.001
NE(^∗^10−9/L)	9.61 ± 4.04	7.33 ± 3.68	10.10 ± 3.97	11.39 ± 3.37	< 0.001
Creatinine (μmol/L)	91.79 ± 50.96	83.02 ± 45.94	96.93 ± 64.17	95.45 ± 38.75	0.074
APTT (sec)	29.68 ± 4.15	29.67 ± 4.72	29.03 ± 3.70	30.32 ± 3.90	0.064
PT (sec)	12.76 ± 2.52	12.73 ± 3.01	12.47 ± 1.33	13.08 ± 2.83	0.183
**Aortic root procedure**					0.200
Ascending aorta replacement	183 (52.89%)	60 (51.72%)	71 (61.74%)	52 (45.22%)	
Bentall’s procedure	138 (39.88%)	47 (40.52%)	40 (34.78%)	51 (44.35%)	
Aortic root repair	23 (6.65%)	8 (6.90%)	4 (3.48%)	11 (9.57%)	
Other	2 (0.58%)	1 (0.86%)	0 (0.00%)	1 (0.87%)	
Concomitant procedures (CABG and valve surgery)	32 (9.22%)	14 (12.07%)	9 (7.83%)	9 (7.76%)	0.430
Lowest nasopharygeal temperature (.C)	24.31 ± 1.54	24.34 ± 1.38	24.40 ± 1.70	24.19 ± 1.53	0.578
Lowest bladder temperature (.C)	25.68 ± 1.67	25.59 ± 1.38	25.93 ± 1.85	25.52 ± 1.72	0.137
**Times**					
Cross-clamp time (min)	115.45 ± 28.84	113.55 ± 28.95	111.22 ± 25.23	121.54 ± 31.21	0.017
CPB time (min)	207.63 ± 50.61	201.17 ± 44.88	206.20 ± 45.47	215.52 ± 59.47	0.091
MHCAT (min)	27.18 ± 9.81	28.28 ± 9.61	27.12 ± 9.62	26.16 ± 10.16	0.258

*The data are shown as mean ± standard deviation, median (Q1, Q3), or *n* (%). BMI, body mass index; LVEF, left ventricular ejection fraction; WBC, white blood cell; NE, neutrophils; APTT, activated partial thromboplastin time; PT, prothrombin time; CABG, coronary artery bypass grafting; CPB, cardiopulmonary bypass; MHCAT, Moderate hypothermia circulatory arrest time; CI, confidence interval, HR, hazard ratio.*

**TABLE 2:** Multivariable cox regression analyses of 90-day postoperative adverse events in patients with acute type A aortic dissection undergoing arch replacement and FET.

	Non-adjusted		Adjust I		Adjust II	
	HR 95% CI	*P*-value	HR 95% CI	*P*-value	HR 95% CI	*P*-value
D-dimer ug/ml per 10 ug/ml	1.28 (1.11, 1.48)	0.001	1.26 (1.09, 1.45)	0.002	1.19 (1.01, 1.41)	0.039
**D-dimer tertile**						
Lowest tertile (T1)	Reference		Reference		Reference	
Median tertile (T2)	2.13 (1.03, 4.38)	0.041	2.01 (0.97, 4.17)	0.058	1.58 (0.73, 3.39)	0.242
Highest tertile (T3)	3.30 (1.66, 6.55)	0.001	3.16 (1.59, 6.29)	0.001	2.41 (1.15, 5.06)	0.019
*P* for trend		0.0004		0.001		0.015

*CI, confidence interval, HR, hazard ratio. The median (range) D-dimer was 0.51 ug/ml (0.33–0.77 ug/m) in the lowest tertile, 1.95 ug/m (1.26–2.28 ug/m) in the median tertile, and 6.27 ug/m (3.16–13.27 ug/m) in the highest tertile. Non-adjusted model adjust for: None. Adjust I model adjust for: age, sex, and BMI. Adjust II model adjust for: age, sex, and BMI; the history of coronary artery disease and cerebrovascular disease, lower-extremity ischemia, acute visceral ischemia, chronic kidney disease; WBC; cross-clamp time, cardiopulmonary bypass time.*

**TABLE 3:** Effects of D-dimer on 90-day postoperative adverse events in each subgroup by multivariable Cox model.

CPB per 10 min	No. of participants	HR (95% CI)	*P*-value	*P* for interaction
**Age tertiles (year)**				0.814
≤43	106	1.14 (0.84, 2.38)	0.197	
44–52	128	1.19 (0.90, 1.58)	0.231	
≥53	113	1.25 (1.04, 1.50)	0.015	
**Sex**				0.831
Male	275	1.25 (1.05, 1.49)	0.011	
Female	72	1.38 (1.07, 1.79)	0.014	
**BMI tertiles**				0.534
≤24	104	1.76 (1.36, 2.27)	<0.0001	
24–27	117	1.12 (0.89, 1.42)	0.332	
≥27	124	1.30 (0.89, 1.88)	0.170	
**Cerebrovascular disease**				0.105
No	327	1.19 (0.99, 1.43)	0.064	
Yes	20	7.03 (1.10, 44.98)	0.039	
**Coronary artery disease**				0.885
No	324	1.26 (1.08, 1.48)	0.004	
Yes	23	1.24 (0.87, 1.76)	0.236	
**Acute visceral ischemia**				0.072
No	339	1.33 (1.15, 1.54)	0.0001	
Yes	8	–&	–&	
**Lower-extremity ischemia**				0.655
No	321	1.31 (1.12, 1.54)	0.001	
Yes	26	1.06 (0.73, 1.56)	0.750	
**eGFR**				0.558
<60	58	1.33 (1.08, 1.65)	0.008	
≥60	289	1.18 (0.95, 1.46)	0.139	

*^∗^Adjusted for age, sex, BMI, the history of coronary artery disease and cerebrovascular disease, lower-extremity ischemia, acute visceral ischemia, chronic kidney disease except the subgroup variable. BMI, body mass index; CABG, coronary artery bypass grafting; CI, confidence interval; HR, hazard ratio. ^&^The model failed because of the small sample size.*

## Results

**FIGURE 1:**
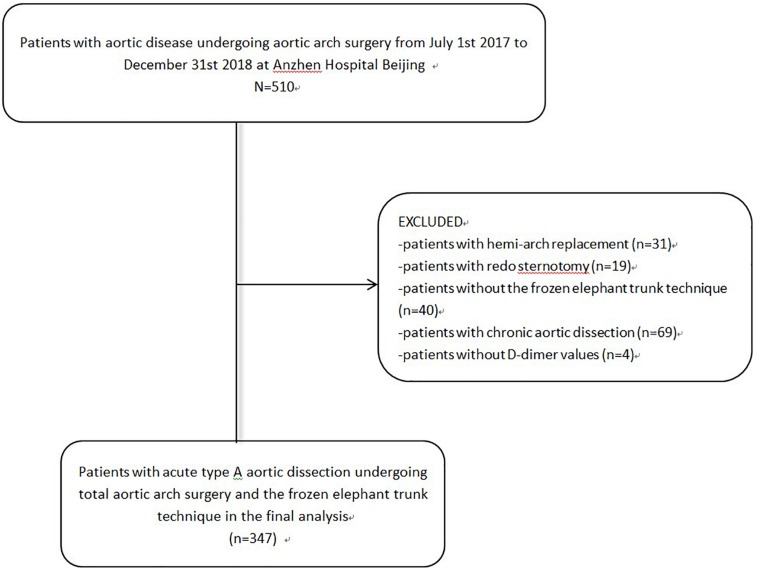
Flow chart.

From July 1, 2017, to December 31, 2018, 510 consecutive patients received aortic arch surgery at the cardiac surgery center in the Beijing Anzhen Hospital. We excluded patients who underwent hemi-arch replacement (*n* = 31), had chronic aortic disease (*n* = 69), underwent redo-sternotomy (*n* = 19), or did not have FET implantation (*n* = 40) (Figure 1).

### Demographic Characteristics

The final analysis included 347 patients with acute type A aortic dissection who underwent total arch replacement and FET implantation (Figure 1). The median D-dimer value was 1.95 ug/mL, with an interquartile range of 0.77–3.16 ug/mL. The baseline demographic, clinical, operative, and perfusion characteristics are presented in Table 1. At admission, the mean age of the 347 patients was 48 years (48.07 ± 10.56), 275 (79.25%) were men. On echocardiography, the mean LVEF was 62.49 ± 5.43 and severe aortic regurgitation was identified in 51 (15.32%) patients at presentation. Moreover, 280 (80.69%) patients had previous hypertension, 13 (3.75%) had previous diabetes mellitus, 58 (16.71%) had chronic kidney dysfunction (CKD), 23 (6.63%) had coronary artery disease, and 20 (5.76%) had cerebrovascular disease. At admission, 26 (7.49%) patients presented with lower-extremity ischemia, 14 (4.03%) with acute cardiac tamponade, 8 (2.31%) with acute visceral ischemia, and 1 (0.32%) with spinal cord injury.

At the time of presentation, no difference was found in the patients’ sex, BMI, LVEF, and clinical status regarding diabetes mellitus, hypertension, coronary artery disease, cerebrovascular disease, the type of aortic root procedure, and concomitant procedures. However, lower-extremity ischemia and CKD were more common among those with higher D-dimer. Further, higher white blood cell counts and longer cross-clamp times were also associated with higher D-dimer.

### D-dimer Levels and 90-day Postoperative Adverse Events in Patients With Acute Type A Aortic Dissection Undergoing Arch Replacement and FET

Sixty-five (18.7%) patients developed 90-day postoperative mortality and PND, consisting of 51 (14.7%) patient deaths and 14 (4.0%) patients with PND.

**FIGURE 2:**
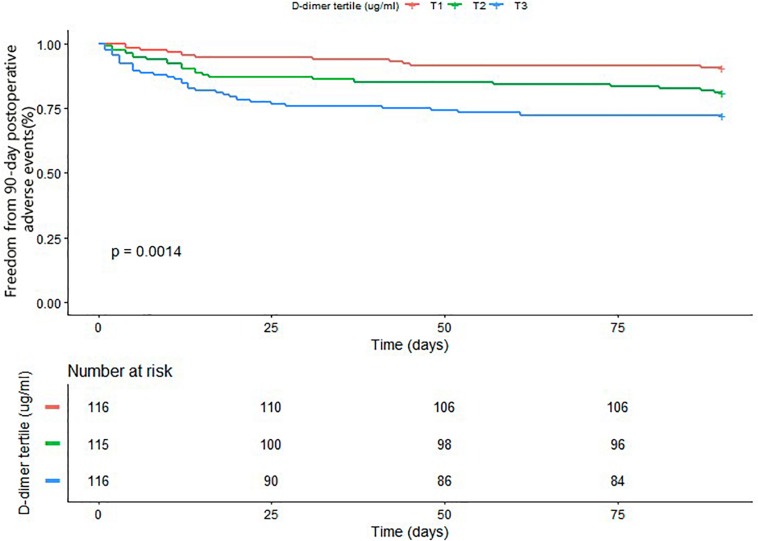
Kaplan–Meier analysis of freedom from 90-day postoperative adverse events based on D-dimer tertile (Log-rank, *P* = 0.0014).

The results of univariate analyses of 90-day postoperative adverse events are summarized in the Supplementary Table. Univariate analyses showed that age, history of coronary artery disease, history of cerebrovascular disease, cardiopulmonary bypass time, cross-clamp time, and D-dimer values were associated with a significant increase in the incidence of 90-day postoperative adverse events. We performed a multivariate Cox regression analysis to further explore D-dimer as a prognostic marker. In the multivariable analysis shown in Table 2, D-dimer level was the independent risk factor for 90-day postoperative adverse events in Model I (HRadj 1.26 per 10 ug/ml increase, 95% CI: 1.09–1.45; *P* = 0.002), after adjusting for age, sex, and BMI. This was also true in Model II (HRadj 1.19 per 10 ug/ml increase, 95% CI: 1.01–1.41; *P* = 0.039) after adjusting for age, sex, BMI, history of coronary artery disease, history of cerebrovascular disease, lower-extremity ischemia, acute visceral ischemia, chronic kidney disease, WBC, cross-clamp time, and cardiopulmonary bypass time. Kaplan–Meier survival analysis (Figure 2) showed a significant difference among patients stratified by D-dimer level tertiles; specifically, D-dimer values in the highest tertile had more 90-day postoperative adverse events compared with the median and lowest tertiles (*P* = 0.0014).

### Subgroup Analysis Between D-Dimer Level and 90-day Postoperative Adverse Events in Patients With Acute Type A Aortic Dissection Undergoing Arch Replacement and FET

To evaluate the potential influence of other factors, a sub-analysis was conducted stratifying patients by age tertiles, sex, BMI tertiles, history of cerebrovascular disease, history of coronary artery disease, acute visceral ischemia, lower-extremity ischemia, and chronic kidney disease, as presented in Table 3. Notably, all subgroups demonstrated a similar overall relationship between D-dimer level and 90-day postoperative adverse events in patients with acute type A aortic dissection undergoing arch replacement and FET.

## Discussion

The results of this study show that serum D-dimer level upon admission is independently associated with 90-day postoperative adverse events in patients with acute type A aortic dissection undergoing arch surgery with FET. For every 10 ug/mL increase in plasma D-dimer concentration, the risk of 90-day postoperative adverse events increased by 19%, after adjusting for multiple factors. To the best of our knowledge, this is the first study to show an association between D-dimer level and 90-day postoperative adverse events in patients with acute type A aortic dissection undergoing arch surgery with FET.

D-dimer is a degradation product of cross-linked fibrin. Elevated D-dimer levels are generally thought to be the result of intravascular activation of the coagulation system and secondary fibrinolysis. Several studies have shown that D-dimer is a good diagnostic test for diverse thrombotic conditions, such as ischemic stroke (Nezu et al., 2018), venous thromboembolism (Faller et al., 2017), pulmonary embolism (van der Hulle et al., 2013), and aortic dissection (Mussa et al., 2016). In addition to its high diagnostic value for thrombotic disease, recent studies have also reported its relationship with short-term and long-term prognosis of these diseases (Kim et al., 2015; Vele et al., 2016; Faller et al., 2017). Additionally, several studies have shown an association in the general population (Di Castelnuovo et al., 2013). For type A aortic dissection, high levels of tissue factors are released from the aortic wall injury, after which marked coagulation activation and subsequent marked enhancement of fibrinolysis occur (Weber et al., 2003). This pathophysiological process elevates serum D-dimer levels in patients with type A aortic dissection. The role of D-dimer in predicting short-term and long-term outcomes in patients with type A aortic dissection has been reported in previous cohort studies (Weber, 2005; Ohlmann et al., 2006; Wen et al., 2013; Huang et al., 2015). Nevertheless, these studies were made in only a limited number of patients. Further, there was significant heterogeneity in the study population, including dissection of different pathological types, as well as dissection patients treated with drugs and surgery. Additionally, for dissection patients undergoing surgery treatment, no surgical-related variables were collected. Thus, there are very few studies that focus on the relationship between D-dimer and 90-day postoperative adverse events in patients with acute type A aortic dissection undergoing arch replacement and FET.

The present study confirmed that D-dimer remains an independent predictor of 90-day postoperative adverse events in patients with type A aortic dissection despite surgical treatment. Although the mechanism of this relationship is not yet clear, some possible explanations may clarify its existence. Firstly, D-dimer concentrations may reflect the anatomical extent of the dissection, which represents the extent of the aortic injury (Weber et al., 2003; Ohlmann et al., 2006). Secondly, plasma D-dimer concentration reflects the volume of intraluminal thrombus, which is the biologically active material that takes part in the evolution of the dissection. It contains neutrophils, released pro-inflammatory cytokines, and proteolytic enzymes, which are associated with the destruction of the aortic wall and the progression of the dissection (Vele et al., 2016). Another study indicated that D-dimer values in mild to moderate traumatic brain injury predict hematoma expansion (Sugimoto et al., 2017). Finally, D-dimer also activates inflammatory cytokines and causes advanced blood coagulation or progression of dissection status. These factors might contribute to the association of high D-dimer concentrations with 90-day postoperative adverse events in patients with type A aortic disease undergoing arch replacement and FET.

In our center, over the past 10 years, although arch replacement with FET under moderate hypothermic circulatory arrest (MHAC) plus ante-grade cerebral perfusion has already become a standard procedure for aortic dissection (Sun et al., 2011; Ma et al., 2013), arch replacement is still a challenging procedure (Leontyev et al., 2016; Mussa et al., 2016). Some previous studies analyzed pre- and intraoperative predictors of early death and PND after arch replacement (Khaladj et al., 2008; Martens et al., 2016; Liu et al., 2017). Liu et al. (2017) found that the history of cerebrovascular diseases was a strong predictor of adverse outcome following arch replacement in 626 consecutive patients in China. Khaladj et al. (2008) found that advanced age and multiple comorbidities (renal insufficiency, coronary heart disease, and reoperation) were risk factors for adverse outcomes in 501 patients undergoing aortic arch surgery. However, these studies did not collect variables of preoperative coagulation status and there was high heterogeneity of selected patients. Considering that preoperative clinical status is a strong risk factor for surgical prognosis in previous studies (Khaladj et al., 2008; Liu et al., 2017), we not only adjusted based on these potential confounding factors in multiple regression models, but we also performed subgroup analysis according to the preoperative clinical status in the current study. Our results also indicate that the relationship between D-dimer and 90-day postoperative adverse events remained unchanged (Table 3).

### Limitations

There are several limitations in this study. First, this study is a retrospective design from a single center, and our results may not be extendable to patients in other centers. Second, we measured D-dimer only on admission, and a series of measurements after arch replacement might be more valuable for evaluation of the association between D-dimer level and 90-day postoperative adverse events. Third, for the treatment of acute type A aortic dissection, aortic arch replacement combined with FET is a preferred choice at our center, while other centers may select more conventional procedures. This might lead to differences in study results. Fourth, this study lacks data on aortic computed tomography angiography (CTA). Because our center is the largest referral center for aortic disease in China, we only referred to the images from the local hospitals, as we can’t repeat the imaging examination for patients with type A aortic dissection given a limited time. Post-operative CTA was also not collected in every patient. The relationship between the level of D-dimer and CTA was not analyzed. Finally, other coagulation factors and tissue factors, such as factor II, V, VII, VIII, IX et al., were not collected because it was not the routine items of the clinical practice in the Beijing Anzhen Hospital (Capital Medical University of Beijing, China).

## Conclusion

D-dimer is easily available in routine medical practice. Our results show that increased D-dimer levels at admission were associated with 90-day postoperative adverse events in patients with type A aortic dissection undergoing arch surgery with FET. This indicates that such high-risk patients deserve close medical monitoring.

## Data Availability Statement

The datasets generated for this study are available on request to the corresponding author.

## Ethics Statement

The studies involving human participants were reviewed and approved by the Beijing Anzhen Hospital. The patients/participants provided their written informed consent to participate in this study.

## Author Contributions

All authors listed have made a substantial, direct and intellectual contribution to the work, and approved it for publication.

## Conflict of Interest

The authors declare that the research was conducted in the absence of any commercial or financial relationships that could be construed as a potential conflict of interest.

## Footnotes

1 http://www.empowerstats.com

2 http://www.R-project.org
