# Corrigendum: Association Between D-dimer and Early Adverse Events in Patients With Acute Type A Aortic Dissection Undergoing Arch Replacement and the Frozen Elephant Trunk Implantation: A Retrospective Cohort Study

**DOI:** 10.3389/fphys.2020.00409

**Published:** 2020-04-28

**Authors:** Tong Liu, Jun Zheng, You-Cong Zhang, Kai Zhu, Hui-Qiang Gao, Kai Zhang, Xiu-Feng Jin, Shang-Dong Xu

**Affiliations:** ^1^Department of Cardiology, Beijing Anzhen Hospital, Capital Medical University, Beijing, China; ^2^Department of Cardiac Surgery, Beijing Aortic Disease Center, Beijing Anzhen Hospital, Capital Medical University, Beijing, China

**Keywords:** D-dimer, predictor, 90-day postoperative adverse events, aortic arch replacement, frozen elephant trunk

An author's name was incorrectly spelled as “Liu Tong.” The correct spelling is “Tong Liu.”

The authors apologize for this error and state that this does not change the scientific conclusions of the article in any way. The original article has been updated.

