# Supplementary material for: Association Between D-dimer and Early Adverse Events in Patients With Acute Type A Aortic Dissection Undergoing Arch Replacement and the Frozen Elephant Trunk Implantation: A Retrospective Cohort Study
Source: Front Physiol. 2020 Jan 21;10:1627. doi: 10.3389/fphys.2019.01627 (PMC6988575; doi:10.3389/fphys.2019.01627)
Supplement: Supplementary file 1 [file Table_1.DOCX]

**Supplement** **Table** Univariate Cox regression analyses of 90-day postoperative adverse events in patients with acute type A aortic dissection undergoing arch replacement and the frozen elephant trunk.

|  | HR (95% CI) | *P* |
| --- | --- | --- |
| Age, y | 1.04 (1.01, 1.06) | **0.003** |
| Sex-female | 1.63 (0.95, 2.78) | 0.075 |
| BMI | 0.99 (0.94, 1.05) | 0.816 |
| Smoking history | 1.07(0.66, 1.74) | 0.778 |
| Diabetes mellitus | 0.74 (0.18, 3.04) | 0.680 |
| Hypertension | 1.33 (0.68, 2.61) | 0.408 |
| Coronary artery disease | 2.62 (1.30, 5.31) | **0.007** |
| Acute cardiac tamponade | 1.83 (0.67, 5.04) | 0.241 |
| Cerebrovascular disease | 2.56 (1.22, 5.37) | **0.012** |
| Acute visceral ischemia | 0.67 (0.09, 4.80) | 0.686 |
| Lower-extremity ischemia | 1.82 (0.87, 3.81) | 0.113 |
| Spinal cord injury | --& | --& |
| Marfan syndrome | --& | --& |
| WBC (g/L) | 1.05 (1.00, 1.11) | 0.064 |
| Aortic root procedure |  |  |
| Ascending aorta replacement | Ref. |  |
| Bentall’s | 1.19 (0.72, 1.96) | 0.496 |
| Aortic root repair | 0.46 (0.11, 1.92) | 0.288 |
| other | --& | --& |
| Concomitant procedures (CABG and valve surgery) | 1.83 (0.90, 3.70) | 0.092 |
| Lowest nasopharygeal temperature (.C) | 0.87 (0.72, 1.04) | 0.134 |
| Lowest bladder temperature (.C) | 0.86 (0.73, 1.01) | 0.061 |
| Cross-clamp time (min) | 1.02 (1.01, 1.02) | **0.001** |
| MHCAT (min) | 0.99 (0.97, 1.02) | 0.533 |
| CPB time | 1.02 (1.01, 1.02) | **<0.0001** |
| D-dimer ug/ml per 10 ug/ml | 1.28 (1.11, 1.48) | **0.001** |
| D-dimer tertile |  |  |
| Lowest tertile (T1) | 1.0 |  |
| Median tertile (T2) | 2.13 (1.03, 4.38) | 0.041 |
| Highest tertile (T3) | 3.30 (1.66, 6.55) | 0.001 |
| P for trend |  | 0.0004 |

BMI: body mass index; WBC: white blood cell; CABG: coronary artery bypass grafting; SACP: selective ante-grade cerebral perfusion; MHCAT: moderate hypothermia circulatory arrest time; CPB: cardiopulmonary bypass; CI: confidence interval, HR: hazard ratio.

& The model failed because of the small sample size.

The median (range) D-dimer was 0.51 ug/ml (0.33-0.77ug/m) in the lowest tertile, 1.95ug/m(1.26-2.28ug/m) in the median tertile, and 6.27ug/m(3.16-13.27ug/m) in the highest tertile.
